# Understanding the early dynamics of the 2014 porcine epidemic diarrhea virus (PEDV) outbreak in Ontario using the incidence decay and exponential adjustment (IDEA) model

**DOI:** 10.1186/s12917-016-0922-2

**Published:** 2017-01-05

**Authors:** Amy L. Greer, Kelsey Spence, Emma Gardner

**Affiliations:** Department of Population Medicine, Ontario Veterinary College, University of Guelph, Guelph, ON N1G 2W1 Canada

**Keywords:** Porcine epidemic diarrhea virus, PEDV, Swine, Pigs, Modeling, Epidemic, Disease dynamics, Forecasting

## Abstract

**Background:**

The United States swine industry was first confronted with porcine epidemic diarrhea virus (PEDV) in 2013. In young pigs, the virus is highly pathogenic and the associated morbidity and mortality has a significant negative impact on the swine industry. We have applied the IDEA model to better understand the 2014 PEDV outbreak in Ontario, Canada. Using our simple, 2-parameter IDEA model, we have evaluated the early epidemic dynamics of PEDV on Ontario swine farms.

**Results:**

We estimated the best-fit R_0_ and control parameter (d) for the between farm transmission component of the outbreak by fitting the model to publically available cumulative incidence data. We used maximum likelihood to compare model fit estimates for different combinations of the R_0_ and d parameters. Using our initial findings from the iterative fitting procedure, we projected the time course of the epidemic using only a subset of the early epidemic data. The IDEA model projections showed excellent agreement with the observed data based on a 7-day generation time estimate. The best-fit estimate for R_0_ was 1.87 (95% CI: 1.52 – 2.34) and for the control parameter (d) was 0.059 (95% CI: 0.022 – 0.117). Using data from the first three generations of the outbreak, our iterative fitting procedure suggests that R_0_ and d had stabilized sufficiently to project the time course of the outbreak with reasonable accuracy.

**Conclusions:**

The emergence and spread of PEDV represents an important agricultural emergency. The virus presents a significant ongoing threat to the Canadian swine industry. Developing an understanding of the important epidemiological characteristics and disease transmission dynamics of a novel pathogen such as PEDV is critical for helping to guide the implementation of effective, efficient, and economically feasible disease control and prevention strategies that are able to help decrease the impact of an outbreak.

## Background

Porcine epidemic diarrhea virus (PEDV) is an alphacoronavirus and has circulated in Asian and European swine since 1971 with documented emergence of the virus into the Iowa swine industry in May 2013 where it rapidly spread to many North American locations [1–6]. The PEDV circulating in North American swineherds is highly pathogenic especially in non-immune, suckling piglets in which it causes watery, hemorrhagic diarrhea and sometimes vomiting with up to 100% morbidity creating a significant economic impact for the swine industry [6–9]. In older, weaned pigs, the disease causes similar symptoms along with anorexia, and lethargy and morbidity remains high (up to 90%), however mortality is significantly reduced with between zero and four percent of animals dying as a result of the infection [6, 9, 10]. It is also thought that the reproductive performance of PEDV infected animals is reduced resulting in smaller litter sizes [11].

Transmission of the virus is primarily via the oral-fecal route specifically due to shedding of infectious virus particles in the feces of infected animals [9]. In addition, transmission can occur via feces contaminated fomites such as boots, equipment, or transportation [12]. Environmental conditions (specifically cold and wet conditions) are believed to play an important role in the ability of the virus to persist within the environment and on fomites suitable for transmission [13]. In addition, data from some regions supports the hypothesis that infectious PEDV contaminated spray-dried plasma; a component of some swine feed products has been another route by which transmission of the virus has occurred [14, 15].

In the province of Ontario, Canada, PEDV emerged in January 2014 however, planning for the possible emergence of the virus in Canada had already begun due to the clinical severity of the disease, rapidity with which it spread widely in the United States in 2013, and the expected devastating economic and emotional toll that the emergence of the disease in Canada would take on the swine industry and Canadian farm families. The dynamics of the 2014 Ontario outbreak were distinct from those observed in the United States, because direct farm-to-farm transmission of the pathogen was not believed to be the route of transmission early in the outbreak [15]. Instead, a common source exposure linked to PEDV contaminated swine feed was believed to be the primary driver of the early outbreak dynamics [15]. Following a voluntary recall of the potentially contaminated feed products, subsequent PEDV cases within the province were believed to be the result of direct transmission between farms/premises.

The prevention and control of PEDV presents significant challenges for the swine industry. Pathogenicity appears to be dependent on a number of important factors including the age of animals affected, type of production system, biosecurity measures in place, the time at which the disease is detected (especially season), the herd size, the health status of the herd (e.g. presence of other co-occurring pathogens such as opportunistic bacterial infections), and the overall immune status of the herd [16–18]. Newborn piglets can be temporarily protected by maternal antibodies and therefore, the intentional exposure of sows to the virus especially within farrow to wean operations is a strategy that is used to quickly achieve some level of protective immunity for the newborn piglets [19].

Mathematical models are important tools for understanding the dynamics of infectious diseases within populations. This understanding contributes to our ability to identify disease prevention and control measures that will have the greatest likelihood of success and also allows for a thorough examination of the uncertainty associated with many epidemic processes. Many disease transmission models are mechanistic in nature and have been built for parsimony in order to present the simplest explanation for the observed data. Models are a mathematical representation of the health states considered to be relevant for the research question and population of interest. Parameter values describe the proposed way in which individuals move between the various health states (e.g. susceptible, infected, recovered). In the case of a mechanistic model, a significant amount of information related to the natural history of the disease, incidence within the population of interest, immune status of the population of interest and contact patterns between individuals and premises is required in order to appropriately parameterize such a model. The Incidence Decay and Exponential Adjustment (IDEA) model was first described in 2013 [20]. The model is not mechanistic in nature but rather is a simple, 2-parameter model that has shown significant promise as a descriptive tool capable of projecting epidemic processes within human populations using limited data [21, 22]. Here we apply the IDEA model to a veterinary infectious disease epidemic, specifically the Ontario PEDV outbreak in 2014. Our objectives were to assess the models ability to: 1) capture the documented patterns of PEDV epidemic growth in Ontario during the 2014 outbreak, 2) provide insight into the impact of different PEDV control measures, and 3) “near-cast” the final epidemic size and duration using only data from early in the outbreak.

## Methods

### Data source

Farm data were obtained from publically available reports published by the Ontario Ministry of Agriculture, Food, and Rural Affairs (OMAFRA) documenting all laboratory confirmed porcine epidemic diarrhea virus (PEDV) in the province of Ontario between January 2014 and January 2015 [23]. Ontario disease surveillance guidelines for PEDV requested that veterinarians visiting swine farms where animals showed signs of diarrhea submit samples to the University of Guelph, Animal Health Laboratory (AHL) for testing. Veterinarians were asked to submit a minimum of three pooled fecal samples (with each pooled sample containing feces from 5 different pigs from different litters or locations). Laboratory confirmation was based on a positive test result from the triplex porcine coronavirus polymerase chain reaction (PCR) test. Testing of diagnostic samples was completed at no cost to clients as part of the OMAFRA/AHL disease surveillance plan. Farms with laboratory confirmed PEDV were encouraged to undertake enhanced biosecurity measures in order minimize the risk to other farms. In addition, herd closures combined with feedback measures to rapidly expose the entire herd and establish herd immunity as quickly as possible were an important component of the response plans. Truck wash protocols (involving sanitation, disinfection, drying and downtime), and manure pumping protocols were put into place in order to minimize the risk of PEDV spread between facilities.

Available data include the date of laboratory confirmation, the county in which the confirmed positive farm was located and the type of swine production (e.g. finisher, nursery, farrow-to-finish, or farrow-to-wean) at the positive site. Due to the severity of the disease, rapid transmission within swine herds, and the critical nature of enhanced biosecurity, a liberal case definition is used whereby, a farm is defined as a confirmed case if the facility has had at least one animal sample confirmed as laboratory positive. The publically available data from OMAFRA do not include any suspected or probable cases and therefore, we do not expect the cumulative incidence to decrease between different time points due to suspect cases being ruled out through laboratory testing or other means.

### The IDEA model

We used a previously described, 2-parameter model to evaluate the early epidemic dynamics of PEDV on Ontario swine farms. The “incidence decay and exponential adjustment” (IDEA) model was first described in 2013 and has since been used to examine the epidemic dynamics of the ongoing Ebola outbreak in West Africa and Middle East Respiratory Syndrome Coronavirus (MERS-CoV) in Saudi Arabia [20–22]. The first model parameter is an exponential growth term. Exponential growth is a function of the basic reproductive number (R_0_) of the pathogen, which is defined as the (average) number of successful transmissions per infected individual within an entirely susceptible population, and the average serial interval [24]. In the case of PEDV and the farm-level nature of the OMAFRA dataset, we modify the definition slightly to consider the farm as the unit of observation. In this case, we define R_0_ as the (average) number of successful farm-to- farm transmissions per infected farm within an entirely susceptible population of swine farms in Ontario. The average serial interval is defined as the time between laboratory confirmation in an index case/farm and laboratory confirmation in a secondary case/farm. The model also includes simultaneous exponential decay in the form of a control parameter (d). This parameter describes the slowing of the disease transmission process that is expected to occur as a result of interventions such as behavior change, increased immunity within the population or enhanced biosecurity. The model is not mechanistic and therefore is unable to identify between different prevention and control measures but rather, as a descriptive tool, permits us to identify the time point at which the epidemic begins to slow.

Previous exploratory work using the IDEA model has demonstrated its utility in describing epidemic processes in circumstances where the reproductive number (R_0_) is low or moderate in size [20]. An important benefit of the model is the ability to fit the model to incidence or cumulative incidence data which is often the most easily accessible public data available in the early stages of an epidemic. In addition, unlike a mechanistic model, the IDEA model does not require extensive assumptions to be made regarding items such as the proportion of the population susceptible to the pathogen yet, appears to generate both epidemic size and duration projections that are in line with observations from a number of high-profile, human infectious disease outbreaks. The model has been shown to be able to detect abrupt changes in the epidemic curve due to disease control activities resulting from interventions. This is done by evaluating the change in the model control parameter (d) between each successive generation of the model.

The structure of the simple, 2-parameter model is as follows:

$$ {\mathrm{I}}_{\mathrm{t}} = \left({\left({\mathrm{R}}_0/{\left(1+\mathrm{d}\right)}^{\mathrm{t}}\right)}^{\mathrm{t}}\right.\kern2.75em (1.0) $$

Where, I_t_ is the number of incident cases in each model generation, R_0_ is the basic reproductive number, d is a control parameter that allows for the decay of disease incidence over time and t is scaled in terms of the generation time. In the absence of any disease control interventions, we would expect the disease to increase over time with cases growing to the power of t. However, when control measures or interventions are implemented in the system, we expect that those controls act on the disease transmission parameter (R_0_) by reducing it over time by a power of t^2.^ Best-fit model parameter values are obtained using maximum likelihood estimation (MLE) by fitting the model to the cumulative incidence data.

### Model analyses

In order to derive an estimate of the generation time for the outbreak (time interval between successive generations of PEDV positive farms), we utilized publically available estimates of the incubation period (approx. 2 days) and the average infectious period (approx. 10 days) [25–28]. This allowed us to derive a mean generation time of seven days for PEDV based on the heuristic that the generation time (T) is the incubation period plus one half of the infectious period. However, due to the highly variable estimates for the incubation period and infectious period combined with a lack of detail regarding the potential connectivity of Ontario swine farms that would create opportunities for farm-to-farm transmission, we conducted sensitivity analyses that examined a range of possible generation times between 7 and 13 days.

Data used for the fitting of the IDEA model included only a subset of all laboratory confirmed PEDV positive farms in Ontario. Specifically, we used cumulative incidence data from February 20, 2014 to April 30, 2014. Twenty cases of PEDV occurred prior to this time period (between January 22, 2014 and February 19, 2014), however these cases have been excluded from our analyses, as it is believed that these initial cases in Ontario were linked to a point source exposure related to PEDV contaminated swine feed [15]. The contaminated feed was identified on February 9, 2014 and subsequently recalled by the manufacturer. As a result, we have excluded all cases prior to February 20^th^, 2014 since direct farm-to-farm transmission is not thought to have been the primary mechanism of transmission for this early time period. In addition, we have excluded five cases that were identified after April 30, 2014 because the time between these cases exceeded the possible generation times suggesting that these were sporadic cases and therefore, cases occurring after April 30, 2014 were also excluded from the analyses.

Initially, we estimated the best-fit R_0_ and d parameters for the farm-to-farm transmission component of the PEDV outbreak (February 20 – April 30, 2014) by fitting the previously described IDEA model to cumulative incidence curves using the R statistical computing environment [29]. The IDEA model is not parameterized in terms of calendar date but rather generation time (epidemic generations) and therefore the observed case data were aggregated based on an estimated generation time of seven days. We also fit the IDEA model to the time series data describing cumulative cases over time in an iterative fashion. We did this by fitting the model to a dataset that included progressively more outbreak generations and comparing model fits using maximum likelihood estimates (MLE) for different combinations of values for R_0_ and d. Lastly, we used the results from the iterative fitting procedure to examine the ability of the IDEA model to predict the projected time course of the observed epidemic using only model based estimates generated from the analysis of a small number of generations.

### Alternate assumptions and sensitivity analyses

We conducted sensitivity analyses to examine alternate assumptions about the possible impact of a generation time that was greater than the base case estimate of seven days (7–13 days). In this case, we refit our model using 8-, 9-, 10-, 11-, 12-, and 13-day generation times. For each model fit, we examined the impact of the updated generation time on the estimated R_0_ and d parameters. In addition, we considered the possible impact of under-reporting in the OMAFRA dataset. In our base case analysis, we assumed no under-reporting of cases. Given the emerging nature of the disease in Ontario as well as the clinical and economic severity of the disease this seemed a reasonable assumption to make. However, an alternative assumption exists which is that the dataset represents only the initial/index case within individual production systems (with some production systems being comprised of many individual farms or premises). To address the possibility of under-reporting in the dataset, we have fit the model examining a range of possible assumptions related to under-reporting (from 0% to 50% under-reporting).

## Results

### Descriptive epidemiology

**Fig. 1:**
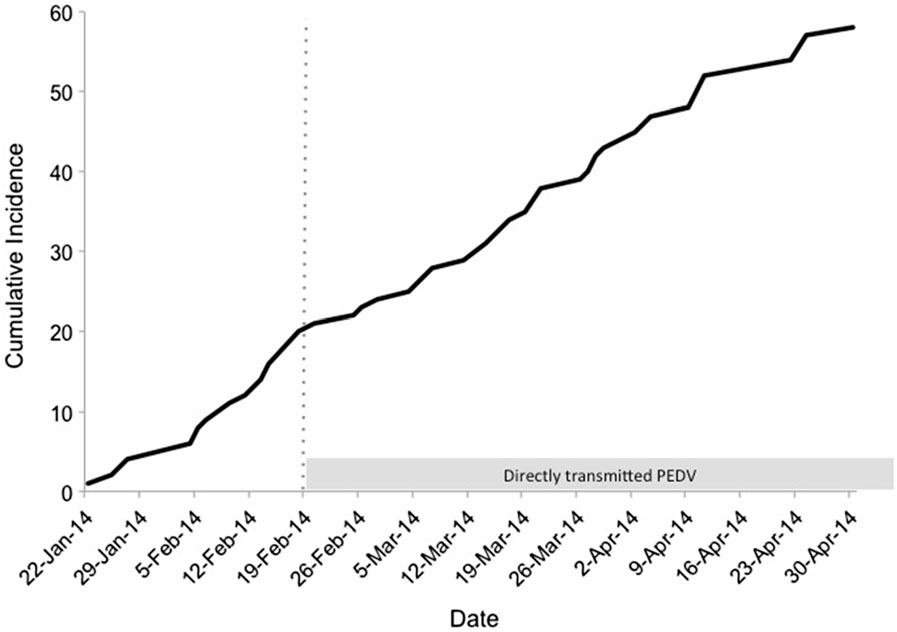
Cumulative incidence data for the Ontario PEDV outbreak in 2014. Cases occurring prior to February 20, 2014 (*dashed line*) are assumed to be the result of a point source exposure through swine feed with cases occurring after February 20, 2014 being the result of direct farm-to-farm transmission

The OMAFRA dataset describes 58 Ontario swine farms that were confirmed to have tested positive for PEDV between January 1^st^, 2014 and April 30^th^, 2014 (Fig. 1). The first confirmed case occurred on January 22, 2014 in Middlesex County and the last confirmed case in the dataset occurred on April 30, 2014 in the same county. All of the confirmed cases were found in southwestern Ontario with the exception of one case on February 14, 2014 that was located in eastern Ontario. Thirty-eight cases occurred during the period of time when transmission is thought to have been the result of direct transmission between farms (after February 19, 2014) rather than through a point source exposure (e.g. swine feed). In the post-recall period, the types of farms represented in the dataset-exhibited differences compared to the pre-recall period. During the pre-recall period, none of the documented cases occurred in finisher herds whereas, in the post-recall period, 61% of the cases occurred in finisher herds.

### Estimation of the Basic Reproductive Number (R_0_) and control parameter (d)

**Fig. 2:**
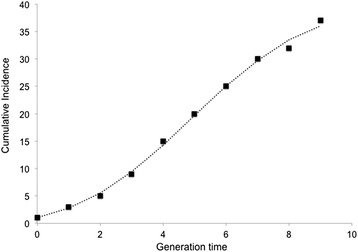
Concordance between model fit (*curve*) and cumulative incidence data for the 2014 Ontario PED outbreak using a mean generation time estimate of 7 days using data from the post-recall time period (after February 19, 2014)

Using case data from the post-feed recall time period (after February 19, 2014), the model showed excellent agreement with the reported outbreak data using all available generations from February 20 to April 30, 2014 and using a generation time estimate of 7 days (Fig. 2). The best-fit estimate for R_0_ was 1.87 (95% CI: 1.52 – 2.34). The best-fit model estimate for the control parameter (d) was 0.059 (95% CI: 0.022 – 0.117). Together these results suggest that the direct transmission period of the outbreak was not highly explosive (R_0_ < 2.5) but was the focus of intensive and effective control measures (*d* > 0.05).

**Fig. 3:**
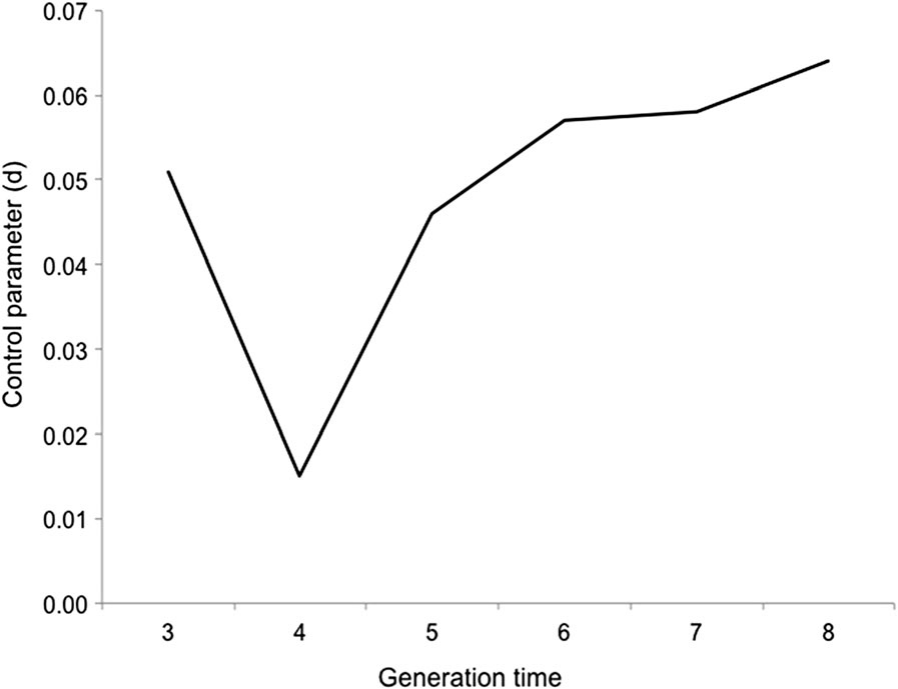
Best fit values for the control parameter (d) at each epidemic generation assuming a 7-day generation time. Values greater than zero are indicative of epidemic slowing. Although the control parameter values were greater than zero from generation three onwards, the magnitude of the value changed over time. The control parameter dropped between generation 3 and 4 indicating epidemic acceleration followed by deceleration of the epidemic growth from generation 4 onwards

Using increasing numbers of successive outbreak generations, we found that there was a significant drop in the control parameter estimate (from 0.051 to 0.0150) in generation 4 (between March 6 and March 13, 2014) and then a return to intensive and effective control in subsequent generations (Fig. 3).

### Short-term outbreak projection

**Fig. 4:**
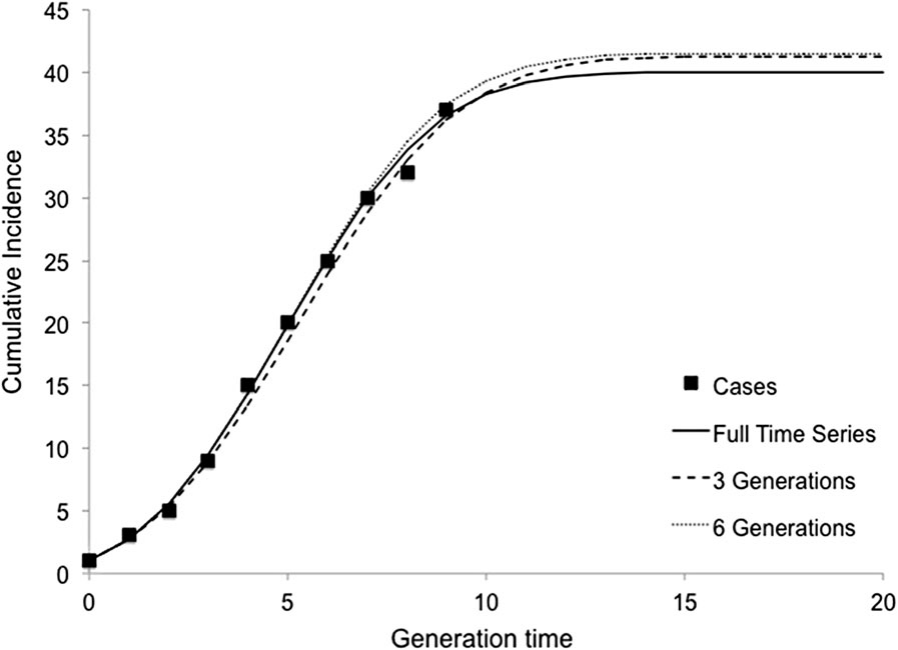
Modeled cumulative incidence based on fitting the IDEA model to three or six epidemic generations assuming a seven day generation time. Squares represent the observed cumulative incidence by generation. The solid line represents the model fit to the entire time series. The dashed lines represent the model fit to only three or six outbreak generations (prior to the outbreak peak). Curves generated from data early in the outbreak are strongly representative of those resulting from fitting to the entire time series

Model-based estimates for R_0_ and the control parameter (d) had stabilized sufficiently by generation three for the IDEA model to be able to project the future course of the outbreak with significant accuracy (Fig. 4). Using only the data available after three generations (March 6, 2014), the model projected that the peak of the outbreak would be reached by generation 12 (May 15, 2014), and that the expected number of cumulative cases would be 41. This is an excellent approximation of the actual observed number of cumulative cases on April 30, 2014 (*N* = 37) predicting the overall final epidemic size within four cases and estimating the end of the outbreak within 15 days. Using six generations worth of data did not significantly improve the predictive ability of the model compared to the estimates available at generation three (Fig. 4).

### Sensitivity analyses and alternate assumptions

**Fig. 5:**
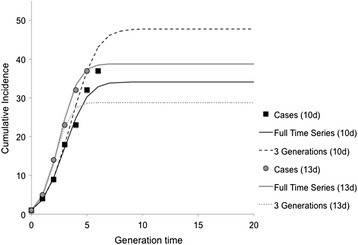
Modeled cumulative incidence based on fitting the IDEA model to three epidemic generations assuming a longer generation time than the base case scenario (10 or 13 days). Squares represent the observed cumulative incidence by generation assuming a 10-day generation time and circles represent a 13-day generation time. Solid lines represent the model fit to the entire time series (black = 10 days, and gray = 13 days). The dashed lines represent the model fit based on only three outbreak generations (prior to the outbreak peak). In this case, curves generated from data early in the outbreak are less representative of those resulting from fitting to the entire time series

Sensitivity analyses were conducted to examine the impact of underestimating the potential generation time for the PEDV outbreak in Ontario. Compared to our base case scenario with a generation time of seven days, increasing the possible generation time (to 10 and 13 days) aggregated the available data into fewer total epidemic generations for the period of time being investigated. As a result, best-fit model parameters for both R_0_ and d were found to increase with increasing generation time (Table 1) representing more explosive epidemic growth but also most intensive intervention and control. However, changing our assumption about the possible generation time resulted in less accuracy when using a small number of epidemic generations to predict final outbreak size and timing (Fig. 5). Using a generation time of 10 days, IDEA model projections of final outbreak size (48 cases) and timing (generation nine, May 21, 2014) when using only data from the first three epidemic generations, overestimated the epidemic by 11 cases and approximately 21 days (Fig. 5). In comparison, using a generation time of 13 days, projections of final outbreak size (29 cases) and timing (generation five, April 26, 2014) when only using data from the first three epidemic generations, underestimated the epidemic trajectory by 8 cases and 4 days (Fig. 5). Suggesting that our initial estimate of 7 days provides the most predictive fit available using minimal, publically available data inputs.

**Table 1:** Sensitivity of IDEA model estimates to alternate assumptions regarding the PEDV generation time and degree of under-reporting of cases

Alternate assumption	R_0_ (95% CI)	d (95% CI)
Base case	1.87 (1.52 - 2.34)	0.06 (0.02 - 0.12)
10-day generation time	3.34 (2.28 - 4.47)	0.19 (0.09 - 0.40)
13-day generation time	4.92 (3.07 – 6.01)	0.30 (0.13 – 0.66)
Outbreak 25% under-reported	2.21 (1.78 – 2.81)	0.16 (0.11 - 0.27)
Outbreak 50% under-reported	2.8 (2.21 – 3.65)	0.12 (0.06 – 0.19)

*CI* confidence interval

We also investigated the potential impact of PEDV underreporting as the epidemic progressed (Table 1). In the case of potential under-reporting, our analyses demonstrate that 25% under-reporting results in an increased R_0_ estimate of 2.21 (95% CI = 1.78 – 2.81) and d estimate of 0.16 (95% CI = 0.11 – 0.27). Increasing the level of underreporting from 25% to 50% further increased the estimated R_0_ to 2.8 (95% CI = 2.21 – 3.65) and reduced d to 0.12 (95% CI = 0.06 – 0.19).

## Discussion

The emergence and subsequent spread of PEDV within the province of Ontario in 2014 represents an important agricultural emergency and the disease remains a threat to the swine industry, and the Canadian economy [15, 30]. Developing an understanding of the important epidemiological characteristics and disease transmission dynamics of a novel pathogen such as PEDV is critical for helping to guide the implementation of effective, efficient, and economically feasible disease control and prevention strategies that are able to help decrease the impact of an outbreak. The ability to obtain short-term projections of epidemic growth and contraction using simple, case-count time series data represents an important capacity that has not been readily available prior to the development of the Incidence Decay and Exponential Adjustment (IDEA) model [20].

Using publically available data from the Ontario Ministry of Agriculture and Rural Affairs (OMAFRA) on the occurrence of PEDV in Ontario in winter 2014 and our previously described IDEA model [20], we have estimated the direct, farm-to-farm, basic reproductive number (R_0_) for the 2014 PEDV outbreak in Ontario. Our estimate of 1.87 (95% CI: 1.52 – 2.34), indicates that the introduction of PEDV into Ontario had the potential to cause an epidemic.

The second parameter of the IDEA model is the control parameter (d). We found that the best-fit control parameter value was 0.06 (95% CI: 0.02 – 0.12). Control parameter values greater than zero indicate that effective control measures were occurring within the province to help slow epidemic growth during the course of the outbreak. In a descriptive sense, an increasing control parameter indicates that the epidemic growth dynamic is slowing [20–22]. There are a variety of possible mechanistic explanations for slowing epidemic growth within the context of the PEDV outbreak including decreased rate of contact between farms (e.g. decreased truck movements), greater farm-level biosecurity, depletion of susceptible farms/premises within the province, environmental conditions which are less favorable for the persistence of the pathogen on vectors of transmission such as trucks and equipment, or any other factor which could act to reduce the force of infection. Despite this limitation, the model does permit the user to identify acceleration or deceleration of the epidemic dynamic based on the available data, including early in the course of an outbreak.

An interesting observation found during the course of this study was that a significant drop in the control parameter (d) was observed in generation four and then the control parameter appeared to rebound in subsequent generations. There is no obvious explanation for this observation however; this period of decreased “control” (and therefore a short period of epidemic acceleration between generation three and four) could be the result of environmental conditions. PEDV is a cold tolerant virus and it is believed that in Ontario, one way that the virus was spread between farms was on PEDV contaminated trucks [12, 13, 31]. Although Ontario implemented rigorous disinfection protocols for vehicles moving between farms, disinfection protocols are less effective in very cold temperatures as it is difficult to properly clean and dry trucks in freezing conditions allowing a cold tolerant virus to persist in the truck environment for longer periods of time and facilitating possible spread between premises [7, 12].

There is significant variability in the documented incubation period and infectious period for PEDV within the published literature [7, 23, 25]. It is thought that some proportion of animals can continue to shed the virus for up to 20–30 days after infection [25]. In this case, the expected generation time would be longer than the seven days that we have used in this study. However, we expect that the highest risk of direct farm-to-farm transmission likely occurs during the initial infection period for a farm/herd when infection rates are still low, clinical symptoms have not yet become widespread, a veterinarian has not yet submitted samples to the laboratory, and therefore enhanced biosecurity measures are not yet in place. This likely describes the first seven days of a farm level outbreak and therefore, this is how we justify our lower limit generation time of seven days. We would expect that as the outbreak becomes more obvious within a specific herd even though shedding may continue to occur for long periods of time, between farm transmission risk is decreased due to enhanced biosecurity.

The to approximate the epidemic final size, epidemic trajectory, and time at which the final size would be reached using only 3 generations worth of simple case count data assuming a generation time of seven days (Fig. 4). For veterinary epidemiologists and others tasked with providing situational awareness and updates to industry during an infectious disease outbreak, the ability to reasonably project forward in time an estimate of the total number of cases expected and the time at which the epidemic is expected to peak would be an incredibly useful application. In this instance, the ability to “near-cast” with some certainty would better allow emergency operations personnel to budget the physical and human resources that would be required to see an outbreak through to completion. Given the uncertainty around the possible generation time estimates, we also examined two alternative scenarios whereby we assumed a longer generation time of 10 or 13 days. In both cases, we found that the model was less accurate at projecting the expected course of the PEDV outbreak using three generations of data assuming a generation time of 10 or 13 days than in the base case that assumed a generation time of seven days (Figs. 4 and 5). This suggests that even without accounting for the possibility of a prolonged duration of viral shedding within some swine, the lower generation time estimate is sufficient to capture the dynamics of the outbreak.

Lastly, it has been proposed that the publically reported data available through OMAFRA may in fact be under-reported by virtue of the fact that for some producers, the laboratory confirmed status documented in the dataset represents only the initial or index farm case within individual production systems (with some production systems being comprised of many individual farms or premises). For instance, a farrow-to-wean farm may test positive for PEDV and some of those infected pigs may move to other farms within the production system causing downstream infections at receiving facilities (e.g. finisher herds etc.). These subsequent downstream farms that become infected may not appear in the OMAFRA dataset as the movement of pigs and subsequent clinical disease in other farms within the same production system occur within a system that is already aware of the PEDV status of the index farm within the production system. The treatment of downstream pigs and enhanced biosecurity would likely be addressed without the submission of additional samples to the laboratory. To address the possibility of under-reporting in the dataset, we examined the potential impact of 25% and 50% under-reporting and found that even if the data represent only a subset of infected farms within Ontario, the model parameter estimates for R_0_ and d remain relatively stable (Table 1) suggesting that the model is not overly sensitive to mild to moderate levels of under-reporting.

As is the case with all epidemiological and mathematical modeling studies, this work has certain limitations, which include the quality of the available data, the specific assumptions that have been made regarding generation time estimates and assumptions related to under-reporting. However, we feel that our findings have demonstrated consistency even when confronted with alternative assumptions and scenarios. It is possible that generation times may differ depending on the type of farm (e.g. farrow-to-weaning vs. farrow-to-finisher). However, for our analysis, the two dominant herd types were finisher herds (61%) and farrow-to-finish herds (21%) suggesting that there was consistency in the type of herds throughout the time period considered which likely minimizes the potential impact of this variability. While it may in fact be the case that the publically reported data available online through the OMAFRA website may underrepresent the true number of PEDV positive farms in Ontario during this time period, the use of publically available data has a clear advantage of allowing for rapid and transparent analysis which would likely not be possible during times of emergency if obtaining data directly from the swine industry was the only way to conduct such analyses.

## Conclusions

Using a simple mathematical model that considers only two parameters, we have demonstrated that the epidemic growth of the 2014 swine PEDV outbreak in Ontario, Canada was to be expected with a reproductive number that exceeded one. However, our analyses also demonstrated that the outbreak began to quickly decelerate (d > 0) as a result of enhanced disease control efforts relatively quickly. Our successful application of the IDEA model to a case study of livestock infectious disease outbreak data is encouraging and suggests that despite some critical differences in the interpretation of the key model parameters and in the individual unit of study (farms/herds/premises vs. individuals), the model can provide useful information for decision-makers in the early stages of an infectious disease outbreak. We encourage the veterinary epidemiology community to continue to assess the usefulness of this simple model in the context of other emerging diseases of veterinary importance. In the case of real-time application and use of the model within an emergency management context, it is our hope that the model may be able to allow for the rapid identification of whether or not interventions are working to control the epidemic spread of the disease.

## Abbreviations

IDEA: Incidence decay and exponential adjustment
OMAFRA: Ontario Ministry of Agriculture, Food, and Rural Affairs
PEDV: Porcine epidemic diarrhea virus

## References

[1] Tian P-F, Jin Y-L, Xing G, Qv L-L, Huang Y-W, Zhou J-Y (2014). Evidence of recombinant strains of porcine epidemic diarrhea virus, United States, 2013. Emerg Infect Dis.

[2] Huang YW, Dickerman AW, Piñeyro P, Li L, Fang L, Kiehne R (2013). Origin, evolution, and genotyping of emergent porcine epidemic diarrhea virus strains in the united states. MBio.

[3] Chen Q, Li G, Stasko J, Thomas J, Stendland W, Pillatzki A (2014). Isolation and characterization of porcine epidemic diarrhea viruses associated with the 2013 disease outbreak among swine in the United States. J Clin Microbiol.

[4] Oldham J. Letter to the Editor. Pig Farming. 1972;10:72–3.

[5] Pensaert M, De Bouck P (1978). A new Coronavirus-like particle associated with diarrhea in swine. Arch Virol.

[6] Stevenson GW, Hoang H, Schwartz KJ, Burrough ER, Sun D, Madson D (2013). Emergence of Porcine epidemic diarrhea virus in the United States: clinical signs, lesions, and viral genomic sequences. J Vet Diagn Invest.

[7] EFSA Panel on Animal Health and Welfare (2014). Scientific Opinion on porcine epidemic diarrhoea and emerging porcine deltacoronavirus. EFSA J.

[8] Song D, Park B (2012). Porcine epidemic diarrhoea virus: a comprehensive review of molecular epidemiology, diagnosis, and vaccines. Virus Genes.

[9] Martelli P, Lavazza A, Nigrelli AD, Merialdi G, Alborali LG, Pensaert MB (2008). Epidemic of diarrhoea caused by porcine epidemic diarrhoea virus in Italy. Vet Rec.

[10] Lin C-N, Chung W-B, Chang S-W, Wen C-C, Liu H, Chien C-H (2014). US-like strain of porcine epidemic diarrhea virus outbreaks in Taiwan, 2013–2014. J Vet Med Sci.

[11] Olanratmanee EO, Kunavongkrit A, Tummaruk P (2010). Impact of porcine epidemic diarrhea virus infection at different periods of pregnancy on subsequent reproductive performance in gilts and sows. Anim Reprod Sci.

[12] Lowe J, Gauger P, Harmon K, Zhang J, Connor J, Yeske P (2014). Role of transportation in spread of porcine epidemic diarrhea virus infection, United States. Emerg Infect Dis.

[13] Jung K, Chae C (2004). Effect of temperature on the detection of porcine epidemic diarrhea virus and transmissible gastroenteritis virus in fecal samples by reverse transcription-polymerase chain reaction. J Vet Diagn Invest.

[14] Dee S, Clement T, Schelkopf A, Nerem J, Knudsen D, Christopher-Hennings J (2014). An evaluation of contaminated complete feed as a vehicle for porcine epidemic diarrhea virus infection of naïve pigs following consumption via natural feeding behavior: proof of concept. BMC Vet Res.

[15] Pasick J, Berhane Y, Ojkic D, Maxie G, Embury-Hyatt C, Swekla K (2014). Investigation into the role of potentially contaminated feed as a source of the first-detected outbreaks of porcine epidemic diarrhea in Canada. Transbound Emerg Dis.

[16] McOrist S. PED - Epidemiology and risk factors for transmision in east Asia. Chicago: Int. Conf. Swine Enteric Coronavirus Dis; 2014. Available from: https://www.aphis.usda.gov/animal_health/animal_dis_spec/swine/downloads/meeting/presentations/24%20-%202%20-%20McOrist.pdf.

[17] Dufresne L, Robbins R. Field experience with porcine epidemic diarrhoea. Am Assoc Swine Veterinarians. 2014;613–16.

[18] Williamson S, Strugnell B, Thomson J, Webster G, McOrist S, Clarke H (2013). Emergence of severe porcine epidemic diarrhea in pigs in the USA. Vet Rec.

[19] Bandrick M, Theis K, Molitor TW (2014). Maternal immunity enhances Mycoplasma hyopneumoniae vaccination induced cell-mediated immune responses in piglets. BMC Vet Res.

[20] Fisman DN, Hauck TS, Tuite AR, Greer AL (2013). An IDEA for short term outbreak projection: nearcasting using the basic reproduction number. PLoS One.

[21] Fisman D, Khoo E, Tuite A. Early epidemic dynamics of the West African 2014 ebola outbreak : estimates derived with a simple two-parameter model. PLOS Curr Outbreaks. 2014. 10.1371/currents.outbreaks.89c0d3783f36958d96ebbae97348d571 PMC4169344 25642358

[22] Majumder M, Rivers C, Lofgren E, Fisman D. Estimation of MERS-coronavirus reproductive number and case fatality rate for the spring 2014 Saudi Arabia outbreak: insights from publicly available data. PLOS Curr Outbreaks. 2014. 10.1371/currents.outbreaks.98d2f8f3382d84f390736cd5f5fe133c PMC4322060 25685622

[23] Ontario Ministry of Agriculture and Rural Affairs. Porcine Epidemic Diarrhea. OMAFRA Website. 2015. Available from: http://www.omafra.gov.on.ca/english/food/inspection/ahw/PED-advisory.html. Accessed 19 Dec 2014.

[24] Vynnycky E, White RG, Fine PEM (2010). An Introduction to Infectious Disease Modelling.

[25] World Organisation for Animal Health (OIE) (2014). Infection with porcine epidemic diarrhoea virus.

[26] Geiger J, Connor J. Porcine Epidemic Diarrhea, Diagnosis, and Elimination. Univ. Minnesota, Coll. Vet. Med. webpage. 2013. p. 1–4. Available from: https://www.aasv.org/aasv%20website/Resources/Diseases/PED/13-05-29PEDWhitePaper.pdf.

[27] Linden J. Porcine epidemic diarrhea. Pig Site. 2014.

[28] Hank Harris D. Porcine Epidemic Diarrhea. Merck Vet. Man. 2013. Available from: http://www.merckvetmanual.com/mvm/digestive_system/intestinal_diseases_in_pigs/porcine_epidemic_diarrhea.html.

[29] R Core Team (2013). R: A language and environment for statistical computing.

[30] MacDougald D (2014). Lessons Learned From PEDV.

[31] Thomas PR, Karriker LA, Ramirez A, Zhang J, Ellingson JS, Crawford KK (2015). Evaluation of time and temperature sufficient to inactivate porcine epidemic diarrhea virus in swine feces on metal surfaces. J Swine Heal Prod.

